# Measurement of Urinary Triclocarban and 2,4-Dichlorophenol Concentration and Their Relationship with Obesity and Predictors of Cardiovascular Diseases among Children and Adolescents in Kerman, Iran

**DOI:** 10.1155/2022/2939022

**Published:** 2022-01-20

**Authors:** Habibeh Nasab, Moghaddameh Mirzaee, Majid Hashemi, Saeed Rajabi

**Affiliations:** ^1^Environmental Health Engineering Research Center, Kerman University of Medical Sciences, Kerman, Iran; ^2^Department of Environmental Health Engineering, Faculty of Public Health, Kerman University of Medical Sciences, Kerman, Iran; ^3^Modeling in Health Research Center, Institute for Futures Studies in Health, Kerman University of Medical Sciences, Kerman, Iran; ^4^Department of Environmental Health Engineering, School of Health, Shiraz University of Medical Sciences, Shiraz, Iran

## Abstract

Exposure to Endocrine-Disrupting Chemicals (EDCs) at an early age can lead to chronic diseases. 2,4-Dichlorophenol (2,4-DCP) and Triclocarban (TCC) are among EDCs that disrupt the endocrine system and alter the body's metabolism. In the present study, the hypothesis that exposure to 2,4-DCP and TCC affects obesity and predictors of cardiovascular diseases was investigated. Fasting Blood Sugar (FBS), Total Cholesterol (TC), Triglyceride (TG), Low-Density Lipoprotein (LDL), High-Density Lipoprotein (HDL (tests were performed on 79 children and adolescents. Also, blood pressure, Body Mass Index (BMI), and BMI z-score were measured to examine the hypothesis. Urinary concentrations of TCC and 2,4-DCP were measured by Gas Chromatography-Mass Spectrometry (GC/MS). Mean concentrations of TCC and 2,4-DCP (*µ*g/L) were higher in obese individuals (5.50 ± 2.35, 0.29 ± 0.13, respectively). After adjusting for possible confounding factors, the results showed an increase in TCC concentration among girls and a decrease in 2,4-DCP among boys with increasing age. The 2,4-DCP concentration among girls increased by 0.007 and 0.01 units with a one-unit increase in Diastolic Blood Pressure (DBP) and FBS, respectively. There was a significant relationship between TCC and TG (Odds Ratio (OR) = 1.02, *p*-value = 0.007), LDL (OR = 1.05, *p*-value = 0.003), and HDL (OR = 0.88, *p*-value = 0.002). There was also a significant relationship between 2,4-DCP and TG (OR = 1.02, *p*-value = 0.002), LDL (OR = 1.12, *p*-value = 0.007), and HDL (OR = 0.92, *p*-value = 0.02). Exposure to TCC and 2,4-DCP can increase some heart risk factors and increase the risk of cardiovascular diseases and obesity. However, to confirm the results of the present study, it is necessary to conduct further studies, such as cohort and case-control studies, with a larger sample size to examine the causal relationships.

## 1. Introduction

One of the serious problems in recent years has been the prevalence of obesity among children and adolescents [1–3]. Obesity leads to many diseases including diabetes, hypertension, and cardiovascular diseases [4, 5]. People who are overweight and obese during childhood are more likely to develop obesity, cardiovascular diseases such as hypertension, hyperlipidemia, and atherosclerosis during adulthood [6]. However, the spread of cardiovascular disease does not begin in childhood and adolescence, but the risk factors for cardiovascular disease often start in childhood and adolescence [7, 8].

These factors include disorders of blood lipid profile, which include Total Cholesterol (TC), Triglyceride (TG), Low-Density Lipoprotein (LDL), High-Density Lipoprotein (HDL, which is one of the most common disorders in cardiovascular disease, especially in obese people. It is well known that there is a potential link between dyslipidemia and obesity, and symptoms of dyslipidemia include decreased HDL and increased TG. Also LDL can affect blood pressure by increasing aldosterone [9, 10]. The prevalence of hypertension in children and adolescents in developing countries has been reported to be between one and five percent through systematic review [11]. Given the rapid increase in obesity in the young population, early detection of risk factors and their prompt treatment are important to prevent or delay the onset of late-stage heart disease complications [9]. The causes of obesity have been widely studied but are not yet well understood [8, 12].

The main causes of obesity are genetic factors, inactivity, diet, and environmental pollutants [13–15]. Environmental pollutants include the role of endocrine-disrupting chemicals (EDCs) (such as phthalates, parabens, triclosan, triclocarban, and diphenols) in the development of chronic diseases. EDCs contain many chemicals used in industry, commerce, agriculture, and pharmacy that have entered the water, air, food, human life, and wildlife [16–18]. They can disrupt the function of the endocrine system and thus be harmful to human health [1, 2, 12, 19]. Some of the factors affecting cardiovascular diseases include age, sex, diet, physical activity, and exposure to EDCs [20, 21]. Exposure to EDCs at an early age can lead to chronic diseases that, if accompanied by improper lifestyle such as inactivity, unhealthy nutrition, increases the risk of chronic diseases [13, 22].

EDCs cause dysfunction of thyroid and sex hormones [16] and dysfunction of the nervous system [23]. They also increase the risk of cardiovascular diseases and obesity [16]. EDCs also play a key role in causing cardiovascular problems [23]. The onset and progression of some of the factors affecting cardiovascular diseases can begin in the critical period of childhood and adolescence [7]. Some EDCs have a long half-life, which is why they have a stable higher level in the environment [24].

2,4-Dichlorophenol (2,4-DCP) is a lipophilic chlorinated phenol [25] from the family of Phenoxyacetic acid herbicides [26, 27], which may be produced by the deformation of 2,4-Dichlorophenoxyacetic acid and triclosan [28]. This compound has high toxicity and resistance to degradation and the bioaccumulation potential of 2,4-DCP is significant [26, 29]. The biological half-life of 2,4-DCP is between 20 and 312 days [30]. Dichlorophenols (DCPs) are considered class B2 (moderate toxicity) herbicides by the World Health Organization (WHO) [26]. The US Environmental Protection Agency (EPA) has identified DCPs as dangerous pollutants [29]. There is an increasing trend in using 2,4-DCP herbicide due to its low cost, and its effectiveness in controlling a wide range of weeds [30].

Triclocarban (TCC) is a carbanilide (3,4,4′-Trichlorocarbanilide) [31]. TCC is an antimicrobial and fat-soluble substance used in a wide range of personal care products, including soaps, toothpaste, shampoos, and detergents [24, 32–34]. Also, environmental pollution by TCC due to its widespread use, population growth, and high environmental resistance has increased its entry into wastewater, sediments, and many water sources [35, 36]. Inadequate removal of TCC in wastewater treatment plants and its high environmental resistance can lead to its accumulation in sludge and sediments, which may enter the food chain if used in agriculture [33, 37].

Animal studies show that TCC can cause dysfunction of sexual and thyroid hormones [38, 39], cause oxidative stress, and biological disorders [24]. Pesticides such as chlorophenols have many side effects on a child's development, including neurological and metabolic outcomes such as diabetes and obesity [40]. Animal studies have also shown that exposure to phenoxy herbicides can lead to lipid peroxidation [26]. 2,4-DCP may disrupt estrogen receptors [41], cell membrane phospholipid structure [42] and cause lipid peroxidation in human red blood cells [28]. TCC and 2,4-DCP enter the body mainly through swallowing and skin [26]. These substances have been observed in blood and urine [25, 43].

There are a limited number of studies showing the relationship between EDCs (phthalates, bisphenol A) with cardiovascular risk factors in children and adolescents in Iran [19, 44–48]. The present study aimed to measure the TCC and 2,4-DCP concentrations and their relationship with predictors of cardiovascular diseases among children and adolescents in Kerman, Iran.

## 2. Materials and Methods

### 2.1. Study Population

The present cross-sectional study was conducted on children and adolescents in Kerman, Iran, in 2020. Random sampling was performed on eligible people.

To calculate the sample size, taking into account the correlation coefficient (*r*) of 0.3, the type I error (*α*) was 0.05, and the test power (*z*_1−*β*_) was 80% and using the following formulas, the sample size was 100, which due to the increase in costs, 79 samples were measured [45].

(1) $$\begin{array}{c} n={\left[\frac{z_{1-\left(\alpha /2\right)}+z_{1-\beta }}{c}\right]}^{2}+3C=\frac{1}{2}ln\frac{1+r}{1-r}. \end{array}$$

The inclusion criteria were having no history of chronic illness, participants aged 6 to 18 years, no long-term use of drugs, and living in Kerman for at least a year. A questionnaire was used to obtain demographic information and the physical activity of participants in the study [19, 44].

According to standard protocols, physical examinations included measurements of height, weight, waist circumference, and blood pressure. The people's height was measured using a meter in a standing position while they were wearing no shoes in a position where three points of the body (heel, back of the head, buttocks) are tangential to the wall. Waist Circumference (WC) was measured using a tape meter in the exhaled position and from the deepest area of the waist between the chest and pelvis. Weight was measured using a digital scale while the person was wearing the lightest clothes. Body Mass Index (BMI) was also calculated using the formula (dividing the weight in kilograms by the height in meters squared). Then, the BMI z score of individuals was calculated by entering age and sex using WHO AnthroPlus software. Blood pressure (diastolic blood pressure (DBP), Systolic blood pressure (SBP)) was measured from the right hand in a sitting position using a digital sphygmomanometer. Suitable cuff was used for children [44, 49, 50].

A total of 2 ml of blood was taken from participants on an empty stomach (no food intake for least 8 hours before the test) to perform the following lipid profile tests: TC, TG, LDL, HDL, and fasting blood sugar (FBS) [9, 44]. The Hitachi 704 automatic analyzer (Hitachi, Tokyo, Japan) was used to determine the serum level of these samples. Urine samples were also taken from the subjects for the creatinine test performed by the Hitachi 704 automatic analyzer [6, 19]. A total of 6 ml of urine sample was kept in glass containers at −20°C until the day of the experiment to measure the TCC and 2,4-DCP concentration [44, 51–53].

### 2.2. Measurement of TCC and 2,4-DCP Concentrations in Urine Samples

First, the urine samples stored in the freezer at −20°C have been melted at room temperature [54]. Analytes were extracted by adding about 2 mL of hydrochloric acid to 5 mL of the urine sample and incubating in a shaking incubator at 70–80°C for one and a half hours. Then 1 mL of tert-Butyl methyl ether (MTBE) solvent was added to it and placed in a vortex shaker for 5 minutes. Afterward, 100 *μ*L of Hexane was added to it in a dry vial.

The sample extracted from the previous step was transferred to a vial for the GC apparatus and 5 *μ*L of the N-Trimethylsilyl-N-methyl trifluoroacetamide (MSTFA) derivatizing reagent and placed in the oven at 50°C for 60 minutes. Then 1 *μ*L of this solution was injected into the gas chromatography-mass spectrometry (GC/MS). Using different column temperature programming of GC/MS and carrier gas flow rate (helium carrier gas with a purity of 99.999 and flow rate 1 mL min^−1^ was used). The best peak separation was achieved. Splitless was used as the GC injection technique [44, 47, 55].

### 2.3. Statistical Analysis

Data analysis was carried out using SPSS (Ver. 22) and STATA (Ver. SE 12). Kolmogorov–Smirnov and Shapiro–Wilk tests were used to examine the data distribution. An independent *t*-test was used to compare the means. Univariate and multiple linear regression analysis was used to investigate the relationship between 2,4-DCP and TCC with the studied variables. Multiple logistic regression analysis was used to compare different 2,4-DCP and TCC tertiles in predictors of cardiovascular diseases and obesity by removing potential confounders.

## 3. Results

Table 1 shows the demographic information of participants. The mean age of the study population was 11.36 ± 3.81 years. Most of the participants were in the age range of 6–11 years (50.6%). The majority of children and adolescents were not exposed to secondhand smoke (81%). Parents of most children and adolescents did not have an academic (69–76%) and made a small income (59.5%). Most of the children and adolescents studied had moderate to high physical activity (38%), using cosmetics (53.2%), and the number of baths less than twice a week (50.6%).

The results of Table 2 show the average concentration of analytes according to the individual's weight. 2,4-DCP was observed in all samples (*n* = 79) but TCC was detected in 94.93% of the samples (*n* = 75). The mean concentration (*µ*g/L) of TCC and 2,4-DCP in obese patients was 5.50 ± 2.35, 0.29 ± 0.13, respectively.

Multiple regression analysis was used to investigate the relationship between analytes concentration and age, physical activity, cosmetics consumption, and the number of baths per week among boys and girls, and results showed no significant relationship between 2,4-DCP and TCC (Table 3).

Table 4 shows the effect of predictors of cardiovascular diseases and obesity on TCC and 2,4-DCP (*µ*g/L) in both crude and adjusted models (adjusted by age, physical activity, BMI, BMI z-score, WC, SBP, DBP, FBS, TC, HDL, LDL, and TG).

The adjusted model showed that the TCC concentration decreased by 0.39 units in girls with increasing age (year). A one-unit increase in BMI increased the TCC concentration to 0.64 units in all subjects. The TCC concentration increased by 0.08 units for a one-unit increase in DBP.

The results also showed that the 2,4-DCP concentration decreased by 0.02 units in boys with increasing age (year). The 2,4-DCP concentration increased 0.007 units for one-unit increasing DBP in girls. The 2,4-DCP concentration in girls was increased by 0.01 units for a one-unit increase in FBS.

Table 5 shows the relationship between 2,4-DCP, TCC (*µ*g/L) with the studied variables. The studied data were divided into three parts (three tertiles) based on 2,4-DCP and TCC variables. The first tertile was considered as a reference. Data in three models (model 1: crude; model 2: adjusted by age and gender; Model 3: adjusted physical activity, age, BMI z-score, WC, BMI, SBP, DBP, FBS, TC, TG, LDL, HDL) were calculated by calculating the odds ratio (OR) with 95% confidence interval.

In Model 2, for a one-unit increase in HDL, the odds of TCC in the third tertiles was 0.88 times that of the first tertile. For a one-unit increase in LDL, the odds of TCC in the third tertiles were 1.05 times that of the first tertile. For a one-unit increase in TG, the odds of TCC in the third tertiles was 1.02 times than the first tertile.

In Model 2, for a one-unit increase in HDL, the odds of 2,4-DCP in the third tertiles was 0.92 times than the first tertile. For a one-unit increase in TG, the odds of 2,4-DCP in the third tertiles was 1.02 times than the first tertile. For a one-unit increase in LDL, the odds of 2,4-DCP in the third tertile was 1.05 times that the first tertile. In Model 3, for a one-unit increase in LDL, the odds of 2,4-DCP in the third tertiles were 1.12 times than the first tertile.

## 4. Discussion

The present study measured the concentration of urinary analytes (TCC and 2,4-DCP) in children and adolescents and investigated their relationship with predictors of cardiovascular diseases and obesity. TCC and 2,4-DCP were observed in the urine samples of most children and adolescents, which indicates that the majority of people are exposed to these analytes.

The present study showed that the 2,4-DCP concentration was higher in children (6–11 years) than adolescents (12–18 years) (Table 4). Children are more exposed to environmental pollutants than adults due to behavioral characteristics, more respiration, and eating more food than our body requirement [22]. 2,4-DCP is a herbicide widely used in many agricultural products to control weeds [26]. 2,4-DCP is easily absorbed through the skin due to its lipophilic properties [25]. Since children have a higher skin-to-body ratio than adults, they may absorb a higher dose of the environmental pollutants [56].

There have been few studies on the relationship between exposure to TCC and 2,4-DCP with predictors of cardiovascular disease and obesity. The results of the present study showed a positive relationship between increased urinary concentrations of 2,4-DCP and TCC with BMI z-score and obesity. The study by Buser et al. showed that BMI z-score, WC, and obesity increase with increasing 2,4-DCP concentration in children and adolescents aged 6 to 19 years [40]. The study by Wei et al. also showed a linear relationship between 2,4-DCP concentration and obesity [57]. The study by Xie et al. demonstrated a positive relationship between exposure to TCC with impaired glucose tolerance (IGT) and type 2 diabetes (T2DM) in women [58].

The present study showed a significant relationship between TCC and 2,4-DCP levels with lipid profiles (TG, HDL, LDL). EDCs can disrupt the metabolism of liver fatty acids, which can affect the blood lipid concentration [59]. Phenolic compounds have been shown to stimulate oxidative changes in living cells that may lead to lipid peroxidation. Lipid peroxidation can be defined as the oxidative degradation of lipids containing any number of carbon-carbon double bonds. Degradation of membrane lipids alters its integrity, fluidity, and permeability, the loss of biological membrane function and the conversion of LDL to nonestrogenic and anti-inflammatory forms. 2,4-DCP induces lipid peroxidation and significantly increases lipid peroxidation in the animal liver [28]. Studies show that exposure to TCC affects glycolipid metabolism in rat liver [43]. TCC also has the potential to inhibit soluble epoxide hydrolase, which is effective in cholesterol synthesis [35, 60].

The present study showed a significant relationship between TCC and 2,4-DCP concentrations with and DBP. TCC and 2,4-DCP can disrupt the endocrine system to some extent by binding to nuclear receptors, interfering with sexual and thyroid hormones [34, 61–63]. Since steroid hormones play a role in regulating blood pressure, for example, estrogen, progesterone, and testosterone receptors in blood vessels relax blood vessels and inhibit vascular smooth muscle contraction mechanisms by stimulating endothelial-dependent mechanisms, it can be assumed that these chemicals may affect blood pressure [64].

In vivo experiments show that TCC has high potency in inhibiting enzyme soluble epoxide hydrolase (sEH). The sEH exerts its cardiovascular effects during processes including vasodilation, antitransmission measures on vascular smooth muscle cells, and anti-inflammatory procedures. This enzyme is also involved in cholesterol synthesis, which proves that TCC can alter the function of biological inflammation, pain, and blood pressure [35, 60, 65].

The present study showed a significant relationship between 2,4-DCP concentration and FBS. Type 2 diabetes is characterized by abnormally high blood glucose levels [66]. EDCs can interfere with insulin secretion and function [67]. By targeting alpha cells in the pancreas, EDCs disrupt molecular signals, which in turn induces glucagon release when blood glucose levels are low, consequently increasing the risk of diabetes [68]. 2,4-DCP as an EDC can affect the expression of estrogen receptors [41]. Experiments on laboratory animals show that 2,4-DCP affects 17*β*-estradiol levels [27]. Estradiol can be a stimulant and promoter of type 2 diabetes (estradiol is associated with changes in glucose homeostasis) [69].

Despite the results of the present study, different EDCs may have different or contradictory impacts. For example, obese people do not always have diabetes or insulin resistance, but there are people who, despite being underweight or having normal weight, suffer from serious metabolic problems such as insulin resistance and type 2 diabetes [66, 70].

## 5. Limitation and Strengths of the Study

The present study has several strengths such as examining the effect of 2,4-DCP and TCC on most predictors of cardiovascular disease and obesity and evaluating the effects of TCC and 2,4-DCP on the sensitive population (children and adolescents) as new research in Iran. The present study has limitations such as low sample size and being a cross-sectional study that requires longitudinal and interventional studies to investigate the effective factors.

## 6. Conclusion

The present study showed that exposure to TCC and 2,4-DCP in childhood and adolescence can be a potential risk factor for the development of cardiovascular disease and obesity indices. In the present study, higher 2,4-DCP and TCC concentrations were observed among children aged 6–11 years and there was a significant relationship between the mean concentration of analytes and weight status. Also, after the removal of possible confounders, we found a relationship between 2,4-DCP and TCC with DBP and lipid profile as well as 2,4-DCP with FBS was observed. Since childhood and adolescence, obesity can lead to health problems in adulthood. Environmental pollution control should be considered as a health priority to prevent noncommunicable diseases. This study could also warn us of stricter regulations to reduce the use of TCC and herbicides such as 2,4-DCP in consumable and agricultural products in developing countries, especially Iran.

## Figures and Tables

**Table 1:** Distribution of variables demographic in the population.

Variables	All *n* (%)
Age groups	
6–11 years	40 (50.6)
12–18 years	39 (49.4)
Mean ± SD	11.36 ± 3.87
Smoker family	
Nonsmokers	64 (81.0)
Smokers	15 (19.0)
Father education	
Illiterate	11 (13.9)
Nonacademic	60 (75.9)
Academic	8 (10.1)
Mother education	
Illiterate	7 (8.9)
Nonacademic	55 (69.6)
Academic	17 (21.5)
Household income (US$/month)	
599≥	47 (59.5)
600≤	32 (40.5)
Physical activity	
Low	19 (24.1)
Moderate	30 (38.0)
High	30 (38.0)
Cosmetic consumption	
Yes	42 (53.2)
No	37 (46.8)
The number of baths (Weeks)	
≤2	40 (50.6)
≥3	39 (49.4)

**Table 2:** - Mean concentration of analytes according to weight status.

Analytes concentration (*µ*g/L)	No. of positives all (*n* = 79)	Sd ± mean				*p*-value
		Total	Underweight/Normal	Overweight	Obese	
TCC	75 (4 < LOD)	4.62 ± 1.93	3.66 ± 0.90	4.66 ± 1.64	5.50 ± 2.35	0.001
2,4-DCP	79	0.23 ± 0.14	0.17 ± 0.10	0.22 ± 0.19	0.29 ± 0.13	0.001

**Table 3:** Association between variables and concentration of urinary analytes (*µ*g/L) s.

Variable	TCC (*µ*g/L)				2,4-DCP (*µ*g/L)			
	Boys		Girls		Boys		Girls	
	*β*	*p*-value	*β*	*p*-value	*β*	*p*-value	*β*	*p*-value
Physical activity (PA) a								
Low	Ref	—	—	—	Ref	—	—	—
Moderate	−0.49	0.55	−0.48	0.54	−0.03	0.53	−0.07	0.31
High	−0.36	0.61	−0.71	0.45	0.02	0.62	−0.06	0.41
Cosmetic consumption^b^								
No	Ref	—	—	—	—	—	—	—
Yes	0.29	0.61	0.98	0.13	—	—	—	—
The number of baths (Weeks)								
≤2	Ref	—	—	—	—	—	—	—
≥3	0.04	0.93	−0.53	0.42	—	—	—	—
	*R* ^2^ = 0.09		*R* ^2^ = 0.23		*R* ^2^ = 0.23		*R* ^2^ = 0.16	

a: Physical Activity: low = less than 5 minutes, moderate = 5 to 30 minutes, high = more than 30 minutes b,c: 2,4-DCP is not used in personal care products.

**Table 4:** The effect of the studied variables on TCC and 2,4-DCP.

Variable	TCC (*µ*g/L)			2,4-DCP (*µ*g/L)		
	Boys	Girls	Total	Boys	Girls	Total
	*β*	*β*	*β*	*β*	*β*	*β*
Age						
Crude	−0.01	−0.05	−0.03	−0.002	−0.008	−0.006
Adjusted	−0.17	−0.39^*∗*^	−0.25^*∗*^	−0.02^*∗*^	0.008	−0.01
BMI						
Crude	0.10	0.23^*∗*^	0.17^*∗*^	0.01^*∗*^	0.006	0.009^*∗*^
Adjusted	0.43^*∗*^	0.82^*∗*^	0.64^*∗*^	0.01	−0.006	0.01
BMI z-score						
Crude	0.25	0.48^*∗*^	0.38^*∗*^	0.02^*∗*^	0.01	0.02^*∗*^
Adjusted	−0.73	−1.60^*∗*^	−1.05^*∗*^	−0.03	0.03	−0.02
WC						
Crude	−0.006	0.03	0.01	0.001	0.00	0.001
Adjusted	−0.01	−0.03	−0.01	0.002	0.00	0.001
SBP						
Crude	−0.02	−0.02	−0.02	0.001	−0.001	4.6
Adjusted	−0.05	0.004	−0.02	0.00	−0.004	−0.002
DBP						
Crude	0.02	−0.05	−0.01	0.00	0.003	0.001
Adjusted	0.08^*∗*^	−0.01	0.02	0.002	0.007^*∗*^	0.003
FBS						
Crude	−0.05	−0.02	−0.03	−0.005	0.003	−0.001
Adjusted	−0.01	−0.009	−0.003	−0.002	0.01^*∗*^	0.00
TC						
Crude	0.02	0.003	0.008	−0.001	0.00	−3.06
Adjusted	0.004	−0.001	0.002	−0.001	0.00	−0.001
HDL						
Crude	−0.05	−0.09^*∗*^	−0.07^*∗*^	−0.003	−0.002	0.003
Adjusted	−0.03	−0.02	−0.02	0.001	−0.001	0.00
LDL						
Crude	0.02^*∗*^	0.01	0.01^*∗*^	0.004^*∗*^	0.001	0.001^*∗*^
Adjusted	−0.003	−0.01	−0.01	0.003	0.002	0.002
TG						
Crude	0.01^*∗*^	0.02^*∗*^	0.01^*∗*^	0.001^*∗*^	0.001	0.001^*∗*^
Adjusted	0.004	0.004	0.001	0.00	0.00	0.00

Adjusted by body mass index (BMI), BMI z-score, waist circumference (WC), Systolic blood pressure (SBP), diastolic blood pressure (DBP), fasting blood sugar (FBS), total cholesterol (TC), High-density lipoprotein (HDL), Low-density lipoprotein (LDL), triglycerides (TG), physical activity, age ^∗^*p*-value ≤ 0.05.

**Table 5:** association among a urinary concentration of analytes and predictors of cardiovascular risk factors.

Variable	TCC^a^ (*µ*g/L)		2,4-DCP^b^ (*µ*g/L)	
	Tertile 2^c^ OR (95% CI)	Tertiles 3^d^ OR (95% CI)	Tertile 2 OR (95% CI)	Tertiles 3 OR (95% CI)
SBP				
Model 1	1.01 (0.96–1.07)	0.97 (0.92–1.02)	1.00 (0.95–1.05)	1.01 (0.96–1.07)
Model 2	1.01 (0.96–1.07)	0.97 (0.92–1.03)	1.00 (0.95–1.05)	1.02 (0.97–1.08)
Model 3	1.04 (0.94–1.14)	0.97 (0.87–1.07)	1.02 (0.92–1.12)	0.99 (0.89–1.11)
DBP				
Model 1	1.01 (0.95–1.07)	0.98 (0.92–1.04)	0.99 (0.93–1.06)	1.02 (0.96–1.08)
Model 2	1.008 (0.95–1.06)	0.97 (0.91–1.04)	0.99 (0.93–1.06)	1.01 (0.95–1.08)
Model 3	0.99 (0.90–1.09)	1.01 (0.90–1.14)	0.99 (0.89–1.11)	1.04 (0.92–1.19)
HDL				
Model 1	0.92 (0.86–0.98)^*∗*^	0.90 (0.84–0.97)^*∗*^	1.00 (0.95–1.05)	0.94 (0.89–1.00)
Model 2	0.91 (0.84–0.98)^*∗*^	0.88 (0.82–0.95)^*∗*^	1.00 (0.95–1.06)	0.92 (0.86–0.99)^*∗*^
Model 3	0.93 (0.84–1.04)	0.93 (0.83–1.04)	1.04 (0.94–1.16)	0.98 (0.85–1.13)
LDL				
Model 1	1.04 (1.01–1.08)^*∗*^	1.05 (1.01–1.09)^*∗*^	1.03 (1.00–1.07)^*∗*^	1.05 (1.02–1.09)^*∗*^
Model 2	1.05 (1.01–1.09)^*∗*^	1.05 (1.01–1.09)^*∗*^	1.03 (1.00–1.07)^*∗*^	1.05 (1.02–1.09)^*∗*^
Model 3	1.01 (0.95–1.06)	0.99 (0.93–1.05)	1.03 (0.97–1.09)	1.12 (1.03–1.22)^*∗*^
TG				
Model 1	1.01 (0.99–1.02)	1.02 (1.005–1.03)^*∗*^	1.00 (0.99–1.02)	1.02 (1.00–1.04)^*∗*^
Model 2	1.01 (0.99–1.02)	1.02 (1.006–1.04)^*∗*^	1.00 (0.99–1.02)	1.02 (1.01–1.04)^*∗*^
Model 3	0.99 (0.97–1.01)	1.00 (0.97–1.02)	1.00 (0.97–1.02)	1.01 (0.98–1.04)
TC				
Model 1	1.01 (0.99–1.04)	1.01 (0.99–1.04)	1.02 (0.99–1.04)	1.00 (0.98–1.03)
Model 2	1.02 (0.99–1.04)	1.01 (0.99–1.04)	1.02 (0.99–1.04)	1.00 (0.97–1.02)
Model 3	1.01 (0.98–1.05)	1.01 (0.98–1.05)	1.01 (0.97–1.04)	0.96 (0.92–1.01)
FBS				
Model 1	1.002 (0.92–1.08)	1.009 (0.93–1.09)	1.02 (0.96–1.09)	1.02 (0.96–1.09)
Model 2	0.98 (0.91–1.07)	1.01 (0.92–1.09)	1.03 (0.96–1.11)	1.03 (0.97–1.11)
Model 3	1.02 (0.92–1.13)	1.06 (0.93–1.20)	1.00 (0.92–1.10)	1.00 (0.92–1.10)

a- The studied data were divided into three parts based on the variable TCC. b- The studied data were divided into three parts based on the variable 2,4-DCP. C, d- Tertile one is referenced Model 1: crude, Model 2: Adjusted by age and gender, Model 3: Adjusted by DBP, SBP, LDL, HDL, TG, TC, FBS, BMI z-score, WC, BMI, age, physical activity ^*∗*^*p*-value ≤0.05.

## Data Availability

The data that support the findings of this study are available upon request from the corresponding author, Majid Hashemi. The data are not publicly available due to their containing information that could compromise the privacy of research participants.
